# Copper management strategies in obligate bacterial symbionts: balancing cost and benefit

**DOI:** 10.1042/ETLS20230113

**Published:** 2023-12-14

**Authors:** Alex Rivera-Millot, Luke B. Harrison, Frédéric J. Veyrier

**Affiliations:** INRS-Centre Armand-Frappier Santé Biotechnologie, Bacterial Symbionts Evolution, Laval, Quebec H7V 1B7, Canada

**Keywords:** bacteria, copper, evolution, host–pathogen interactions

## Abstract

Bacteria employ diverse mechanisms to manage toxic copper in their environments, and these evolutionary strategies can be divided into two main categories: accumulation and rationalization of metabolic pathways. The strategies employed depend on the bacteria's lifestyle and environmental context, optimizing the metabolic cost-benefit ratio. Environmental and opportunistically pathogenic bacteria often possess an extensive range of copper regulation systems in order to respond to variations in copper concentrations and environmental conditions, investing in diversity and/or redundancy as a safeguard against uncertainty. In contrast, obligate symbiotic bacteria, such as *Neisseria gonorrhoeae* and *Bordetella pertussis*, tend to have specialized and more parsimonious copper regulation systems designed to function in the relatively stable host environment. These evolutionary strategies maintain copper homeostasis even in challenging conditions like encounters within phagocytic cells. These examples highlight the adaptability of bacterial copper management systems, tailored to their specific lifestyles and environmental requirements, in the context of an evolutionary the trade-off between benefits and energy costs.

## Copper, a toxic yet essential metal…

Since the emergence of life on Earth, metals have played a vital role in numerous biological processes. Iron was initially favored by bacteria due to its bioavailability [1], but ∼2.7 billion years ago, the increased presence of oxygen [2], triggered by the emergence of photosynthetic organisms, brought about a significant change [3]. This led to the oxidation of soluble iron into an insoluble form, limiting its availability, setting the stage for other metals soluble in their oxidized state, including copper, and the evolution of numerous diverse metal-based metabolic processes which have become crucial.

Copper is utilized by numerous enzymes involved in various essential metabolic pathways, such as electron transport [4,5], nitrogen metabolism [6,7], degradation of aromatic compounds [8], and oxygen-related reactions. It also plays a critical role in the cytochromes c oxidase of the respiratory chain [9,10], thus contributing to the generation of the proton motive force, the energy source of ATP synthase [11]. However, the evolutionary adoption of copper came with concomitant toxic metabolites. Copper can induce oxidative stress (reactive oxygen species, ROS) by generating highly reactive hydroxyl radicals (OH˙) through the Fenton-like reaction [12–15]. These OH˙ radicals react swiftly with surrounding molecules including proteins, lipids, and nucleotides. Copper is directly or indirectly responsible for other stresses. It is responsible for the formation of reactive nitrogen species (RNS) [14,16]. It can also displace other metals complexed within proteins, leading to their malfunction, particularly in proteins containing iron-sulfur clusters [17]. Finally, copper can also disrupt protein folding [18] and induce disulfide bridge formation [19,20], both of which may impair protein functions, leading to cellular toxicity.

## Copper at the heart of prokaryote–eukaryote interactions

In the environment, some organisms such as amebae, have exploited the toxic properties of metals, particularly copper, for preying on bacteria through phagocytosis. One such mechanism that allows them to subsequently kill their prey is metal poisoning [21]. Phagocytosis is deeply conserved throughout the evolutionary of eukaryotes and has become a crucial component of the immune system of metazoans, including humans. Inflammatory cascades trigger the import of copper in phagocytic cells into phagolysosomes to kill bacteria [22,23]. In the case of humans, the transporter Ctr1 facilitates the import of copper from the extracellular environment [24], which is then managed by the chaperone Atox1 [25,26]. Atox1, in turn, transfers the metal to ATPase ATP7A, which exports cytoplasmic copper into the phagolysosome [22,27]. This metal intoxication works synergistically with other bactericidal factors such as ROS [28,29], RNS [30,31], and indirectly, reactive carbonyl species (RCS) [32–35].

## Defense system accumulation to confront various stresses

**Figure 1.**
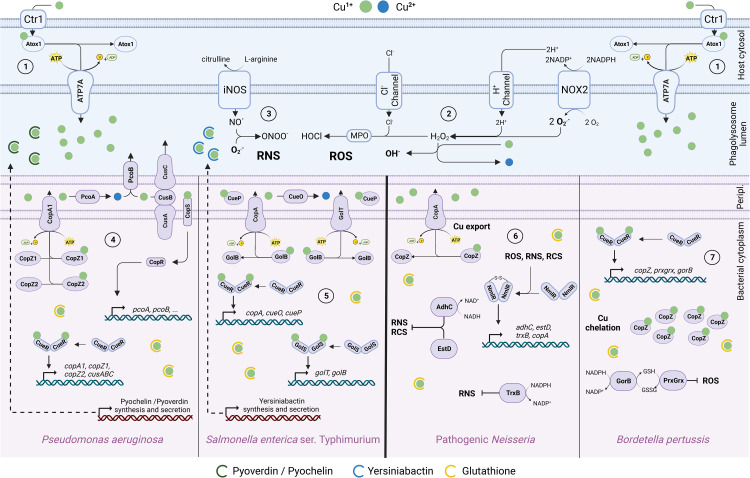
Copper defense mechanisms and adaptations to copper-related stress induced by phagocytic cells. These cells, such as macrophages and neutrophils, generate various forms of stress using copper in the phagolysosome to kill engulfed bacteria. (1) The Ctr1 transporter imports copper into the cytosol, and then the Atox1 chaperone transfers it to ATP7A at the phagolysosome membrane for export into the lumen. (2) NADPH oxidase (NOX2) uses the electron potential of NADPH to produce O2**^.−^** in the phagolysosome lumen. This superoxide anion allows the formation of hydrogen peroxide and certain RNS. Myeloperoxidase produces HOCl using H_2_O_2_. (3) Inducible nitric oxide synthase (iNOS) produces nitric oxide, which leads to various RNS. RCS are produced indirectly by the reaction of ROS and RNS with the organic molecules of the bacterium. The diversity of copper management strategies is depicted in the lower panels. Some bacteria accumulate various homeostasis systems: (4) *P. aeruginosa* possesses two sensor systems, CopRS and CueR, which trigger the expression of disparate copper defense mechanisms. Furthermore, this bacterium can secrete certain molecules such as pyoverdine and pyochelin to buffer copper in the extracellular environment. Other bacteria accumulate redundant systems: (5) *S. enterica* has two closely related regulators, CueR and GolS, of the MerR-type; these regulators control the expression of ATPases and chaperones for copper export and detoxification by chelation. The synthesis and secretion of yersiniabactin also contributes to increased copper tolerance. In contrast, other bacteria exposed to different stresses have rationalized these mechanisms and retain only a minimalist system. (6) Pathogenic *Neisseria* detect reactive species (RS) through the NmlR regulator via disulfide bridge formation. This regulator induces the expression of three RS detoxification genes (*adhC*, *estD*, *trxB*) as well as that of the CopA ATPase copper exporter. (7) *Bordetella pertussis* detects excess copper via the CueR regulator, leading to the production of the CopZ protein capable of detoxifying copper by chelation, as well as two proteins (PrxGrx, GorB) for ROS detoxification utilizing a glutathione redox cycle (reduced: GSH, oxidized: GSSG). In both cases, a single signal triggers multiple responses. The red locii and the dotted arrows represent regulatory and metabolic pathways not detailed here but which play a role in copper tolerance.

Prokaryotic organisms interacting with phagocytic cells have evolved and refined through natural selection strategies for copper detection, tolerance, defense, and detoxification [36] (Figure 1). Yet certain fundamental metabolic processes, like low molecular mass thiols such as glutathione, ubiquitous in all organisms, aid in maintaining cytoplasmic redox balance and limiting copper ion state transitions and their effects [37–39]. Among well studied bacteria, various lifestyles and copper homeostasis strategies have been observed. Bacteria capable of living both free in the environment and inside a host have accumulated a diversity of copper defense mechanisms, some of which exhibit partial redundancy. These mechanisms include systems such as HME-type RND (e.g. CusABC), multicopper oxidases, ATPases, and chelation mechanisms [40]. For example, *Pseudomonas aeruginosa* possesses, in addition to CusABC and PcoAB systems, two independently regulated CopA-type ATPases and two distinct CopZ-type chaperones [41,42]. These different systems are finely regulated by two regulators, CopRS [43], which senses periplasmic copper, and CueR, which senses cytoplasmic copper [41]. Indirect defense mechanisms are also present in *P. aeruginosa*, such as the secretion of pyoverdine and pyochelin, which reduce the toxicity of various metals [44]. Additionally, the repression of copper import proteins like OprC has been observed [45,46]. Other organisms, including *Staphylococcus aureus*, even have mobile genetic elements carrying copper resistance operons, similar to those observed in the context of antibiotic resistance [47,48]. *Salmonella enterica* serovar Typhimurium survives in various environments including the phagolysosome before invading macrophages. This bacterium has two copper ATPases, and a multicopper oxidase CueO (also known as CuiD), but unlike other *Enterobacteriaceae*, *S. enterica* lacks a CusABC-type copper export system [49]. Rather, it has evolved a chelation-based detoxification strategy, involving copper chaperones GolB [50], Csp3 [51], and especially CueP [49,52]. CueP is a periplasmic copper chaperone that largely compensates for the loss of CusABC in terms of copper tolerance [49]. CueP and CusABC appear to have a similar role in metal resistance *in vitro*. However, CueP is more effective in the context of surviving phagocytosis [49]. Despite the accumulation of copper homeostasis systems, some of which are redundant, *S. enterica* appears to have opted for the rationalization of one of these systems by replacing the complex CusABC system with a sole chaperone, CueP. Additionally, *S. enterica* produces and secretes yersiniabactin, a siderophore capable of chelating excess copper the environment [53] and, in the context of an infection, acting as virulence-associated superoxide dismutase mimic [54]. These bacteria have accumulated specific copper defense mechanisms to cope with a range of stresses. In contrast with these examples, there are bacterial genera with an extremely restricted environmental niche. For example, *Neisseria* and *Bordetella pertussis* live almost exclusively on their host's mucosa [55,56]. This highly specific lifestyle appears to be associated with a reduced and optimized genome content which includes specific adaptations for copper homeostasis.

## Copper homeostasis controlled by reactive nitrogen species: the case of *N. gonorrhoeae*

The majority of *Neisseria* species are obligate symbionts that predominantly colonize mucosae of mammalian hosts [57]. Among them, *Neisseria meningitidis* [58] and *Neisseria gonorrhoeae* [59] have been extensively studied due to their pathogenic potential. These bacteria primarily adopt a mucosal-associated extracellular lifestyle with limited intracellular survival capabilities [56]. Analysis of the *N. gonorrhoeae* genome suggests a minimalist copper homeostasis system. This bacterium possesses only the copper chaperone CopZ and the ATPase CopA [40]. Interestingly, this bacterium lacks of the CueR regulator [60], which typically controls the expression of the *copA* and *copZ* genes in gram negative bacteria. Instead, the ATPase is part of a regulon of four genes under the control of the NmlR regulator [60,61]. Comparative genomics suggests that this system is conserved in pathogenic *Neisseria* [62,63]. NmlR is present in several other pathogenic bacteria that colonize the mucosae of their hosts, such as *Haemophilus influenzae* [32,35] and *Streptococcus pneumoniae* [64]. NmlR is involved in regulating the response to ROS, RNS, and/or RCS, depending on the organism. In the case of *N. gonorrhoeae*, this regulator appears to respond to RNS. NmlR controls the expression of genes including *adhC* [65–67], *estD* [67,68], *trxB* [69], and the *copA* ATPase gene. Djoko et al. [60], who described this unique regulatory mechanism, demonstrated a deficiency in intracellular survival in the CopA ATPase mutant. They suggested that this NmlR regulon, which detoxifies RNS, ROS, and copper, is beneficial for survival during phagocytosis, as these three stressors have bactericidal properties. It appears that evolution has led to a rationalization of copper homeostasis mechanisms to cope with specific stresses (Figure 1).

## Defense against oxidative stress under the control of copper: the case of *B. pertussis*

The *Bordetella* genus serves as a model system for studying the evolution of copper homeostasis despite the fact that most *Bordetella* species lack copper defense systems such as CusABC [40,70]. This genus includes bacteria adapted to various environments, with some closely associated with their hosts [71]. For instance, *Bordetella bronchiseptica* can cause respiratory infections in mammals but is able to survive outside the host and resist phagocytosis by both amebas and macrophages [72,73]. Conversely, *Bordetella pertussis*, an obligate symbiont armed with multiple virulence factors, lives primarily on the surface of the human respiratory epithelium [55,74] and has lost many non-essential metabolic pathways [75], including those related to copper [40,76] (Figure 1).

Although *B. bronchiseptica* and *B. pertussis* share several genes related to copper homeostasis, but transcriptomic analyses have revealed that copper defense systems CopI-PcoA-PcoB and CopA are inactive in *B. pertussis* [76]. This bacterium has evolved by deleting portions of its genome and disrupting genes through sequence insertions, particularly IS481, present in several dozen copies [75,77,78]. This evolutionary scenario suggests an ongoing genomic reduction of copper homeostasis systems in *B. pertussis*, as the bacterium is no longer exposed to copper-related stress in the environment and has only retained and refined mechanisms to evade copper-utilizing phagocytic cells [79,80], the last remaining source of copper stress in its niche.

While *B. pertussis* has reduced its copper defense arsenal [40], transcriptomic studies have identified a single remaining system regulated by the copper-sensitive regulator CueR. This system consists of the chaperone CopZ and two proteins involved in detoxifying oxidative stress, a glutathione-dependent peroxidase, and a glutathione reductase [76]. Although the ATPase CopA exporter is inactive due to a sequence insertion, CopZ plays a role in passive detoxification by binding free copper. Even though the other two proteins are not traditionally involved in copper tolerance, they benefit from a more wider dynamic range of regulation through the CueR regulator [76]. This system is specific to situations where copper is present in significant quantities, such as in the phagosome of macrophages, presumably exploiting the fact that in this context, ROS are also used as bactericidal factors [22]. Deletion mutants of this system show a significant decrease in survival after phagocytosis [76]. Outside of these conditions the system is repressed, thus avoiding unnecessary energy expenditure through the synthesis of non-essential proteins during the extracellular multiplication of *B. pertussis* on respiratory mucosa [23,36]. It is interesting to note that the repression of copper importation mechanisms also plays a role in metal tolerance in *B. pertussis*. This bacterium does not secrete siderophores/chalcophores capable of buffering copper but possesses a complex regulatory mechanism, using the CruR protein, leading to strong repression of the TonB-dependent transporter BfrG, hypothesized to be involved in copper import [81]. Therefore, this bacterium has rationalized its copper homeostasis mechanisms by losing non-essential genes and merging the remaining regulon with an oxidative stress defense mechanism (ROS).

## Two strategies: accumulation or rationalization

The evolution of copper homeostasis mechanisms is directly linked to the lifestyle of bacteria. The strength of this relationship is even more pronounced at the host–bacteria interface. Free-living generalist bacteria subjected to environmental stresses have evolved an accumulation of a diverse and/or redundant set of systems to cope with varying conditions. In contrast, specialist bacteria exploiting narrow niches will rationalize their homeostasis mechanisms to both reduce diversity and redundancy to optimize the benefit-to-cost ratio.

## Abbreviations

RCS: reactive carbonyl species
RNS: reactive nitrogen species
ROS: reactive oxygen species
